# Autophagy Activation is Associated with Neuroprotection in Diabetes-associated Cognitive Decline

**DOI:** 10.14336/AD.2018.1024

**Published:** 2019-12-01

**Authors:** Yanqing Wu, Libing Ye, Yuan Yuan, Ting Jiang, Xin Guo, Zhouguang Wang, Ke Xu, Zeping Xu, Yanlong Liu, Xingfeng Zhong, Junmin Ye, Hongyu Zhang, Xiaokun Li, Jian Xiao

**Affiliations:** ^1^Molecular Pharmacology Research Center, School of Pharmaceutical Science, Wenzhou Medical University, Wenzhou, Zhejiang, China; ^2^The Institute of Life Sciences, Wenzhou University, Wenzhou, Zhejiang, China; ^3^Department of Anesthesia, The First Affiliated Hospital, Gannan Medical University, Jiangxi, China

**Keywords:** Diabetes-associated cognitive decline (DACD), Autophagy, Hippocampus, β-amyloid, Apoptosis

## Abstract

Autophagy is a lysosome-dependent cellular catabolic mechanism that mediates the turnover of dysfunctional organelles and aggregated proteins. It has a neuroprotective role on neurodegenerative diseases. Here, we hypothesized that autophagy may also have a neuroprotective role in diabetes-associated cognitive decline (DACD). In current study, we found that db/db mice display cognitive decline with inferior learning and memory function. The accumulation of β-amyloid_1-42_ (Aβ_1-42_), which is a characteristic of Alzheimer’s disease (AD), was markedly higher in the prefrontal cortex (PFC), cornu ammon1 (CA1), and dentate gyrus (DG) areas of the hippocampus in db/db mice. Moreover, BDNF and microtubule associated protein 2 (MAP2) levels were lower in the hippocampus of db/db mice. However, there was no noticeable differences in the level of apoptosis in the hippocampus between control (CON) mice and db/db mice. Markers of autophagy in the hippocampus were elevated in db/db mice. The expression levels of ATG5, ATG7, and LC3B were higher, and the level of P62 was lower. An autophagy inhibitor, 3-MA, and ATG7 siRNA significantly reversed the activation of autophagy *in vitro*, which was accompanied with a higher level of apoptosis. Taken together, our current study suggests that diabetes is associated with cognitive decline, and activation of autophagy has a neuroprotective role in DACD.

Type 2 diabetes (T2D) is a serious chronic metabolic disorder that adversely affects multiple organs due to its long-term complications, and the brain is one of its major targets. As early as 1922, it has been demonstrated that diabetes leads to cognitive dysfunction[1]. Compared to non-diabetic people, patients with T2D perform slightly worse on a range of cognitive tasks[1,2]. To facilitate research into this field and strengthen recognition of this disorder, Mijnhout proposed a new term, diabetes-associated cognitive decline (DACD), in 2006[3]. There is a strong association between Alzheimer’s disease (AD) and T2D[4-6]. A 9-year study showed that the risk of developing AD was 65% higher in people with diabetes than in non-diabetic controls[7]. Results from longitudinal studies also demonstrated that the rate of cognitive decline in patients with T2D is up to two times faster than that those without diabetes[8]. On the other hand, previous studies have shown that diabetic mice demonstrated an accumulation of β-amyloid (Aβ), excessive phosphorylation of tau, neuron loss, and low brain-derived neurotrophic factor (BDNF) in the cortex or hippocampus, which are characteristics of AD[9,10]. Thus, it is crucial to give particular attention to DACD in common degenerative diseases of the central nervous system (CNS).

The autophagic process plays a fundamental role in cellular homeostasis and “housekeeping” mechanisms [11,12]. It participates in organelle turnover or eliminates damaged proteins and organelles under conditions of stress. There are three distinct classes of autophagy: microautophagy, chaperone-mediated autophagy (CMA), and macroautophagy [13]. Macroautophagy begins with the entrapment of material within a double-membrane vesicle called the autophagosome, which fuses into the endolysosomal system to deliver its cargo [13-15]. Microtubule-associated protein 1 light chain 3 (MAP1LC3/LC3) and autophagy-related genes (ATGs) are involved in autophagosome formation [16,17]. P62 (also known as SQSTM1) is a protein associated with autophagosomes, and it is degraded in lysosomes after autophagosomes fuse with lysosomes [18,19]. Macroautophagy affects developmental processes as well as neuronal degeneration and death.

To date, the mechanism of DACD is still unclear. Most research in this field involves three main topics: 1) Oxidative stress leads to changes in hippocampal synaptic plasticity and the destruction of calcium homeostasis in neurons[20]; 2) The high permeability of the blood brain barrier and cerebral microvascular damage [21]; 3) Hyperglycemia directly causes neuronal damage, which leads to mitochondrial dysfunction [22]. However, little is known about the role of autophagy during DACD. Autophagy has been implicated in many neurodegenerative diseases [23,24], including AD [15,17], Parkinson disease (PD) [25], Amyotrophic lateral sclerosis (ALS) [26], and Huntington’s disease [27]. It has been reported that autophagy is involved in the aggregation of AD-related proteins (Aβ and tau) through an increased accumulation of autophagic vesicles in the cerebral cortex [28]. DACD and AD mice have similar behavioral, cognitive, and pathological characteristics [6]. Additionally, the existence of abnormalities during the autophagic process was also described in diabetic patients and animals, which indicate that autophagy may play a key role in DACD formation.

In the present study, we have confirmed that diabetes induced cognitive decline, and observed that autophagy was activated in the hippocampus of db/db mice and PC12 cells cultured in high glucose (HG). There was no significant difference in the level of apoptosis between db/db mice and control mice, indicating that autophagy may play a neuroprotective role during DACD.

## MATERIALS AND METHODS

### Animal experiments and tissue preparation

Eight-week-old male C57BL/BKS-db/db mice and their age-matched non-diabetic mice (control mice, CON) were purchased from Shanghai SLRC Laboratory Animal Co., Ltd. (Shanghai, China) and housed in a specific pathogen-free (SPF) animal experiment center. All experimental procedures for animal studies were approved by the ethics committee of Wenzhou Medical University and were performed in accordance with the Guide for the Care and Use of Laboratory Animals. Animals were housed at 22? with a 12:12 hour light/dark cycle and were given water and a standard mouse diet. After 6 weeks, animals performed behavioral tests. Then, they were anesthetized with 10% chloral hydrate (3.5 mL/kg) and perfused via cardiac puncture initially with 0.9% saline solution. For immunofluorescence and TUNEL assay, the brain was rapidly detached and embedded in O.C.T. compound (Changzhou, Jiangsu, China) for frozen sectioning. Then, it was cut in 5 μm sections using a cryostat (Leica Microsystems Wetzlar GmbH, Hesse-Darmstadt, Germany). For hematoxylin & eosin (H&E) staining and immunohistochemistry, animals were perfused with 4% paraformaldehyde (PFA) in 0.1 M PBS following the saline solution perfusion. Then, the brain was rapidly detached and post-fixed by immersion in 4% PFA for 24 hr. After dehydration by alcohol and transparentizing by dimethylbenzene, tissues were embedded in paraffin. The thickness of paraffin section was 5 μm. For Western blot analysis, the hippocampus was separated from the brain after perfusion with 0.9% saline solution and rapidly stored at -80?.

### Morris water maze

The test was performed in a circular pool with a diameter of 120 cm and a height of 40 cm (Jiliang, Shanghai, China). It was filled with opaque water colored with milk powder and maintained at a temperature of 26 ± 1?. Using a hidden circular platform, the training was carried out with six blocks that consisted of three 60-second trials separated by 20-minute inter-block intervals. During the training, the platform remained in the same location relative to the distal cues in the room. For each trial, mice were placed in the water at different start locations (E, S, W, and N) that were equally spaced from each other and were offset from the goal location by 45°. One hour following the sixth block, the hidden platform was removed, and the mice were scored during a 60 s probe trial. They were scored for latency to reach the goal and for memory recall, which was determined by crossing over the previous platform location. Another probe trial was performed 24 hr after training to assess memory consolidation and memory retrieval.

### TUNEL staining

TUNEL staining was performed using the ApopTag Fluorescein Direct In Situ Apoptosis Detection Kit (Roche, Basel, Switzerland). According to the standard protocol, the frozen sections were fixed by precooled acetone for 15 min and washed with PBS three times. Then, these sections were incubated with 20 μg/ml proteinase K working solution for 15 min at 37?. The slides were then rinsed with PBS three times, which was followed by incubation with the TUNEL reaction mixture for 1 h at 37?. After rinsing with PBS three times for 5 min, sections were treated with 4’, 6-diamidino-2-pheny-lindole (DAPI, Beyotime, Shanghai, China) for 5 min at room temperature and mounted with aqueous mounting medium. The results were imaged using a Nikon ECLIPSE Ti microscope (Nikon, Tokyo, Japan).

### Immunofluorescence staining

For brain sections, the frozen sections were fixed in precooled acetone for 15 min, washed with PBS three times, and then blocked for 30 min at 37? with 5% BSA. Then, sections were incubated with antibodies against LC3B (1:400, Abcam, Cambridge, Britain) or microtubule associated protein 2 (MAP2) (1:200, Abcam) at 4? overnight. Alexa Fluor 647 (1:1000, Abcam) was used as a secondary antibody. Nuclei were labeled with DAPI. For cultured cells, cells on a coverslip were washed 3 times with PBS, fixed in 4% PFA for 30 min, and blocked with 5% BSA for 30 min at 37?. Then, the cells were incubated with the primary antibody LC3B at 37? for 2 hr. The Alexa Fluor 488 secondary antibody was applied at a 1:1000 dilution for 2 hr. The images were captured by confocal laser microscopy (Nikon, Tokyo, Japan).

### Hematoxylin & Eosin (H&E) staining and Immunohistochemistry

The paraffin sections were dewaxed and hydrated before H&E staining. After dewaxing and hydration, H&E staining was performed and eventually observed under a light microscope. For immunohistochemistry, the sections were incubated in 3% H_2_O_2_ for 15 min and 80% carbinol for 30 min, and then they were incubated in blocking solution for 1 hr at 37?. Subsequently, the sections were incubated at 4? overnight with the following primary antibodies: ATG5 (1:200, Bioworld Technology, Shanghai, China), ATG7 (1:200, Bioworld Technology), and Aβ_1-42_ (1:400, Abcam). After washing with PBS three times, the sections were incubated with horseradish peroxidase-conjugated secondary antibodies for 2 hr at 37?, and then they were treated with 3, 3-diaminobenzidine (DAB). The results were imaged using a Nikon ECLPSE 80i (Nikon, Tokyo, Japan).

### Western blot analysis

The hippocampus was separated from the brain soon after the mice were sacrificed, and it was stored at -80? for Western blot analysis. For protein extraction, the tissue was homogenized in lysis buffer containing protease inhibitor cocktail (10 μl/ml, GE Healthcare Biosciences, PA, USA). Then, the complex was centrifuged at 12,000 rpm, and the supernatant was obtained for the protein assay. PC12 cells were lysed in lysis buffer with protease and phosphatase inhibitors. The extracted protein was quantified with BCA reagents. The protein was separated on a 10% or 12% gel and transferred onto a PVDF membrane (Bio-Rad, Hercules, CA, USA). The membrane was blocked with 5% milk in TBST (TBS with 0.05% tween 20) for 0.5 hr and incubated with primary antibodies in TBS for 2 hr at room temperature or overnight at 4?. After it was washed with TBST 3 times, the membrane was treated with horseradish peroxidase-conjugated secondary antibodies (1:3000) for 1 hr at room temperature. Signals were visualized by ChemiDocXRS+Imaging System (Bio-Rad). GAPDH (1:5000, Bioworld) was used as an internal control. All experiments were repeated in triplicate with the use of independently prepared tissue. The densitometric values of bands on Western blots were obtained by Image J software and were subjected to statistical analysis.

### PC12 cell culture and treatment

PC12 cells were purchased from the Cell Storage Center of Wuhan University (Wuhan, China). PC12 cells were cultured in Dulbecco’s Modified Eagle Medium (DMEM, Invitrogen, Carlsbad, CA) supplemented with 10% fetal bovine serum (FBS, Invitrogen) and antibiotics (100 units/ml penicillin, 100 μg/ml streptomycin). They were incubated in a humidified atmosphere containing 5% CO_2_ at 37?. Either 30 mM glucose or mannitol was added to the HG group or the osmotic control, respectively. The cells were treated with or without 5 mM 3-methyladenine (3-MA; Sigma-Aldrich, St. Louis, MO, USA) to inhibit autophagy activation.

### Transfection of ATG7 small interfering RNA (siRNA)

The cells were seeded (5×10^5^ cells/well) in 6-well plates and transfected with siRNA using Opti-MEM media (Invitrogen). Briefly, 100 pmol of ATG7 siRNA (si-ATG7, Cell Signaling Technology) was mixed with Opti-MEM media. Separately, 5 μl Lipofectamine 2000 reagent (Invitrogen) was mixed with Opti-MEM, and then this was combined with the siRNA mixture for 25 min at room temperature. This mixture was then added to each well containing cells and medium and incubated for 4-6 hr. After 24 hr, they were then changed to antibiotic-free DMEM. The cell media was changed to antibiotic-free media prior to the addition of siRNA.

### Fluorescence activated cell-sorting (FACS) analysis

The cells were cultured at a density of 2×10^5^ cells per well in growth medium for 24 hr in 6-well plates. The cells were treated with HG with or without autophagy inhibitor 3-MA or si-ATG7. Annexin V assays were performed using the Annexin V-FITC Apoptosis Detection Kit (Becton Dickinson, San Jose, CA). The cells were washed twice with cold PBS and resuspended with binding buffer before the addition of Annexin V-FITC and propidium iodide (PI). Cells were vortexed and incubated for 15 min at room temperature before analysis using a FACS Calibur flow cytometer (BD Biosciences) and FlowJo software (Tree Star, San Carlos, CA).

### Statistical analysis

Data are expressed as the mean ± SEM. Statistical significance was determined using Student’s t-test in instances of two experimental groups. For more than two groups, statistical evaluation of the results was performed using a one-way analysis-of-variance (ANOVA) test, followed by Tukey’s post hoc test. Statistical significance was accepted when *p*<0.05.

**Figure 1.**
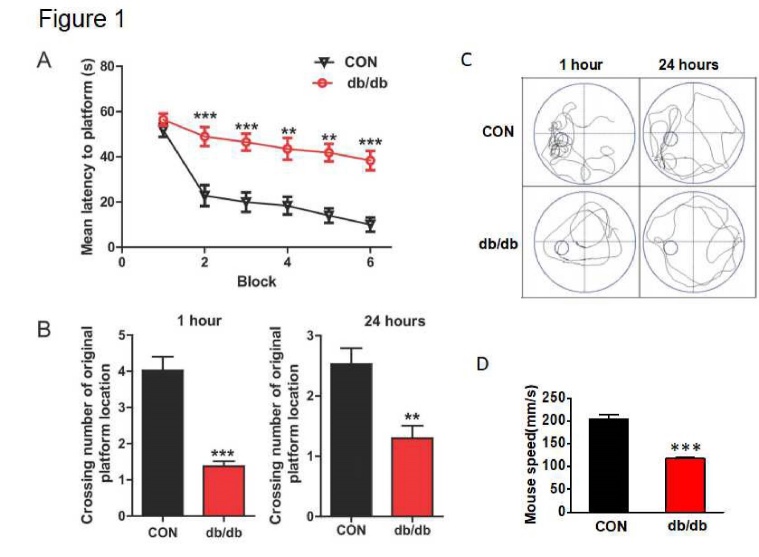
Diabetes resulted in cognitive decline with inferior learning and memory function. (A) The learning curve of training during six blocks in the Morris water maze test of CON mice and db/db mice. (B) Number of crossings over the original platform location in CON mice and db/db mice at 1 hr and 24 hr after training. (C) Representative swimming track of CON mice and db/db mice at 1 hr and 24 hr after training. (D) Swimming speed of CON mice and db/db mice. CON: control. **p < 0.01 *vs.* CON, ***p < 0.001 *vs.* CON, and N = 10.

**Figure 2.**
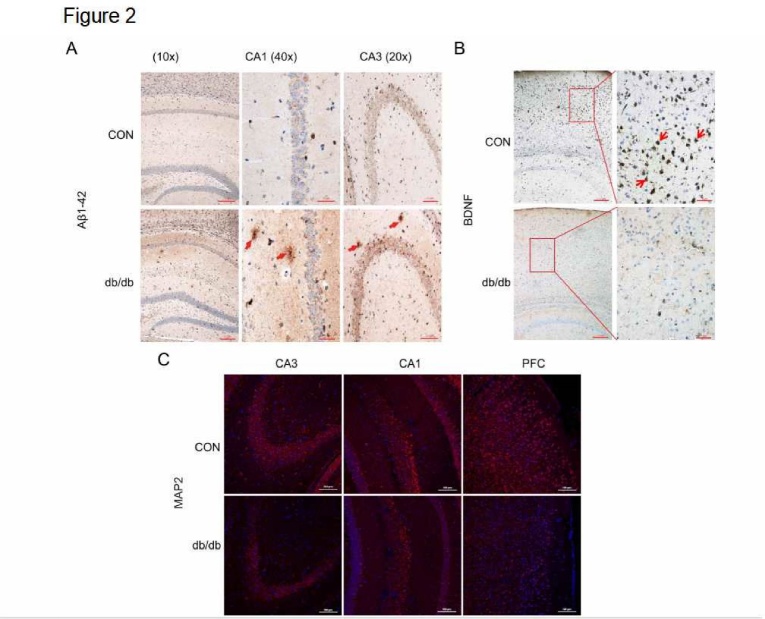
Diabetes induced Aβ_1-42_ accumulation and downregulated the expression of BDNF and MAP2. (A-B) The immunohistochemical staining of Aβ_1-42_ and BDNF in CA1, CA3, and PFC of the hippocampus. (C) The immunofluorescence staining of MAP2 in CA1, CA3, and PFC. CON: control, PFC: prefrontal cortex, CA1: cornu ammon1, and CA3: cornu ammon3Scale bar = 50 μm, and N = 4.

## RESULTS

### Diabetes resulted in cognitive decline with inferior learning and memory function

All mice were trained to learn how to locate the platform throughout six blocks. As shown in Fig. 1A, there is a significant difference in their latency to reach the platform during the six training blocks. db/db mice took longer to reach the platform from block 2 to block 6, when compared to the mice in CON group. After 1 hr of training, the platform was removed, and mice were tested on a probe trial. db/db mice had fewer number of crossings over the platform position compared to the mice from CON group (*p*<0.001). After 24 hr, memory retention of the platform location on the probe trial was worse for db/db mice, as indicated by fewer platform crossings (Fig. 1B and C, *p*<0.01). In addition, the swimming track during 1 hr and 24 hr after training indicate that db/db mice had worse memory compared to mice from the CON group. The swimming speed of db/db mice was also significantly lower than that in the CON group (Fig. 1D).

### Diabetes induced accumulation of Aβ_1-42_ and downregulated the expression of BDNF and MAP2

Amyloid accumulation is one of the most common characteristics of AD[9,10]. Thus, we investigated if DACD induces amyloid accumulation. As shown in Fig. 2A, almost no Aβ_1-42_ immunoreactivity was observed in the hippocampal CA1 and CA3 in mice from the CON group, but the signals were markedly more intense in the hippocampus of db/db mice. BDNF contributes to the induction of synaptogenesis, spine density, and postsynaptic receptor expression in neurons, and its expression is significantly suppressed in the brain of AD patients. Thus, we detected the expression of BDNF. As shown as Fig. 2B, BDNF was normally expressed in mice from the CON group, but its expression was markedly lower in db/db mice. Moreover, we identified a significant reduction of MAP2 immunoreactivity in the hippocampus of db/db mice when compared to mice from the CON group (Fig. 2C). These results indicate that BDNF and MAP2 expression are attenuated by diabetes.

**Figure 3.**
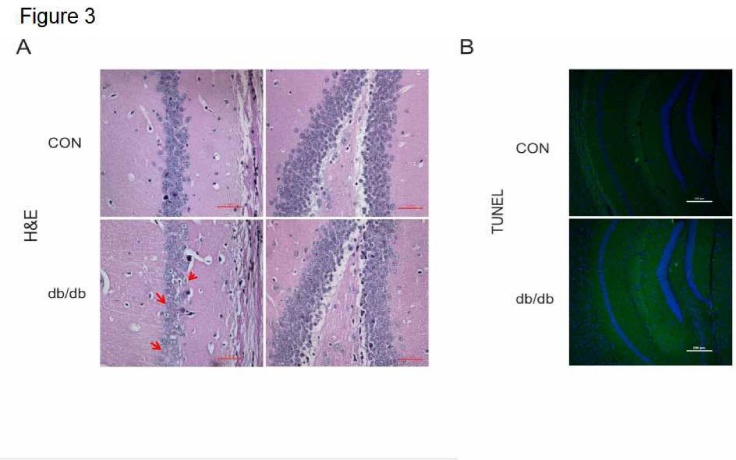
The morphological structure and level of apoptosis in the hippocampus in diabetes. (A) H&E staining of CA1 and DG. (B) TUNEL staining of the hippocampus in CON mice and db/db mice. CON: control. Scale bar = 50 μm, and N = 4.

### The morphological structure and level of apoptosis in the hippocampus of diabetic mice

Animal models of AD show necrotic changes in hippocampal neurons. In the current study, we found that hippocampal CA1 and DG exhibited a clear hippocampus structure in mice from the CON group. They displayed normal cell morphology, large round or oval-shaped nuclei, evident nucleoli, and clear nuclear membranes (Fig. 3A). In the db/db group, neuronal cells were arranged sparsely, and the cell boundaries were blurred (Fig. 3A). However, there was no visible difference on pyknosis when compared to mice from the CON group (Fig. 3A). Moreover, we also detected the level of cell apoptosis using the TUNEL assay. We found no obvious neuronal apoptosis in the hippocampus of db/db mice compared to mice from the CON group (Fig. 3B).

### Autophagy was activated in the hippocampus of db/db mice

**Figure 4.**
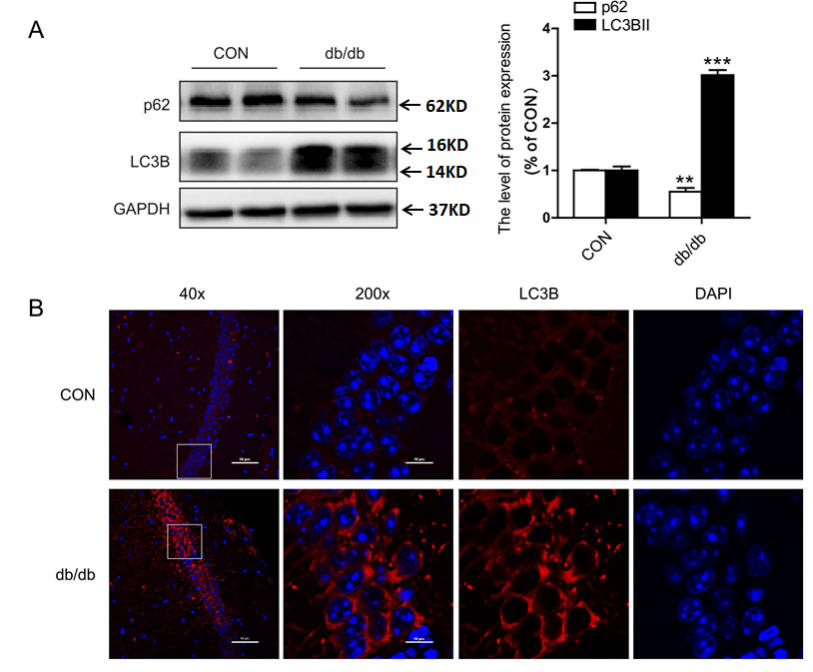
The expression levels of LC3B and P62 in the hippocampus. (A) Western blot and quantitative analysis of LC3B and P62 expression. (B) Immunofluorescence staining of LC3B in CA1. CON: control. Scale bar = 50 µm and 10 µm. **p < 0.01 *vs.* CON, ***p <0.001 *vs.* CON, and N = 6.

**Figure 5.**
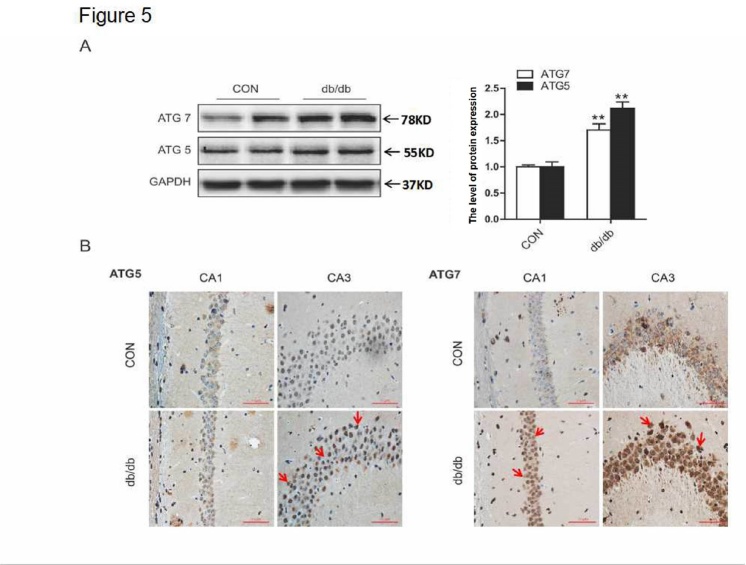
The expression levels of ATG5 and ATG7 in the hippocampus. (A) Western blot and quantitative analysis of ATG5 and ATG7. (B) Immunohistochemical staining of ATG5 and ATG7 in CA1 and CA3. CON: control. Scale bar = 50 µm. **p < 0.01 *vs.* CON, and N = 6.

The conversion of LC3 from the soluble form (LC3-I) to the autophagosome-associated form (LC3-II) and the degradation of P62 (also known as SQSTM1) imply an increase of autophagic activation [16,17]. In the current study, we investigated the expression level of autophagic markers, LC3B and P62. In db/db mice, expression of LC3B-II was upregulated, and P62 was downregulated (Fig. 4A). Consistent with results from Western blot analysis, the positive signals of LC3B were more robust in the CA1 region of db/db mice group (Fig. 4B). Moreover, the expression levels of ATG5 and ATG7 in the hippocampus of db/db mice were significantly higher when compared to mice from the CON group (Fig. 5A and B). Taken together, these data suggest that autophagy was activated in the hippocampus of db/db mice.

### HG treatment increased the expression of autophagy markers in PC12 cells

**Figure 6.**
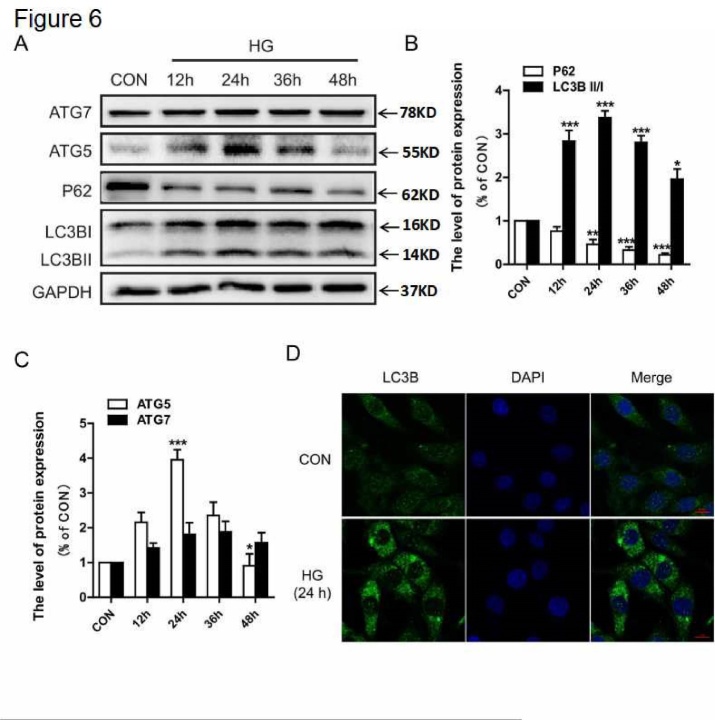
HG treatment increased the expression of autophagy markers in PC12 cells. PC12 cells were cultured in HG (30 mM) conditions for 12, 24, 36, and 48 hr. (A) Western blot analysis of ATG7, ATG5, P62, and LC3B expression. (B) Quantitative analysis of LC3B and P62. (C) Quantitative analysis of ATG5 and ATG7. (D) Immunofluorescence of LC3B in PC12 cells. CON: control, HG: high glucose. Scale bar = 50 μm, *P < 0.05 *vs.*CON, **P < 0.01*vs.* CON, ***P < 0.001 *vs.* CON, and N = 3.

Next, we further determined the level of autophagy under high glucose (HG) conditions *in vitro*. We found that LC3B-II was significantly upregulated from 12 hr to 48 hr in cultured PC12 cells, and this was especially prominent at 24 hr (Fig. 6A and B). The level of P62 was downregulated from 12 hr to 48 hr (Fig. 6A and B). Moreover, the expression levels of ATG5 and ATG7 were increased beginning at 12 hr, and the increase of ATG5 expression was robust at 24 hr but vanished at 48 hr (Fig. 6A and C). Therefore, we chose 24 hr as the treatment time with HG. We also detected the immunofluorescence of LC3B. Notably, we found a marked induction of LC3B immunoreactivity with HG treatment (Fig. 6D). These data indicate that HG increases the expression of autophagy markers in PC12 cells.

### 3-MA treatment inhibited HG-mediated autophagic activation, resulting in the induction of apoptosis

**Figure 7.**
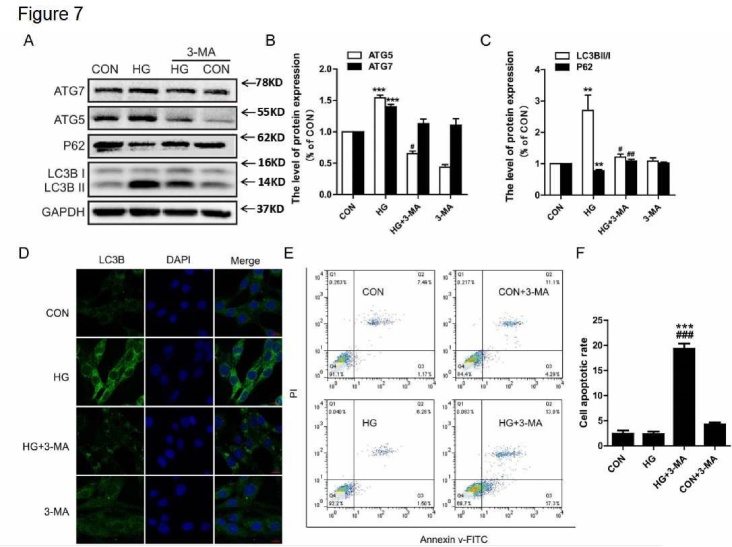
3-MA treatment inhibited HG-mediated autophagic activation, resulting in the induction of apoptosis. (A) Western blot analysis of ATG7, ATG5, P62, and LC3B expression. (B) Quantitative analysis of ATG5 and ATG7. (C) Quantitative analysis of LC3B and P62. (D) Immunofluorescence staining of LC3B in PC12 cells. (E) Cells were collected and stained with annexin V-FITC/propidium iodide and detected by flow cytometry. The lower right panel indicates the apoptotic cells. (F) The level of apoptosis in PC12 cells. CON: control, HG: high glucose. Scale bar = 50 μm, **P < 0.01 *vs.* CON, ***P < 0.001 *vs.* CON, #P < 0.05 *vs.* HG group, ##P < 0.01 *vs.* HG group, ###P < 0.001 *vs.* HG group, and N = 3.

Thus far, our results have indicated that hyperglycemia activated autophagy in the hippocampus, but it did not affect cell death or apoptosis. Therefore, we further investigated the role of autophagy in neuronal survival under HG conditions *in vitro*. In the current study, PC12 cells were treated with HG and treated with or without 3-MA for 24 hr; 3-MA specifically blocks the formation of the autophagosome. We observed that 3-MA inhibited the upregulation of ATG5, ATG7, and LC3B-II caused by HG, and it also induced the expression of P62 (Fig. 7A-C). The results of LC3B immunofluorescence showed that HG increased the level of LC3B puncta, and this was attenuated by 3-MA treatment (Fig. 7D). Next, we further investigated the level of apoptosis in PC12 cells under HG conditions with or without 3-MA for 24 hr. As shown in Fig. 7E and F, HG or 3-MA alone did not noticeably affect the rate of apoptosis. In contrast, HG treatment with 3-MA had a remarkable pro-apoptotic effect compared to the HG group (19.34 ± 1.78% versus 2.35 ± 0.85%, Fig. 7E and F). These data indicate that autophagy might play a neuroprotective role under HG conditions.

### ATG7 siRNA treatment abolished the neuroprotective role of autophagy under HG conditions

**Figure 8.**
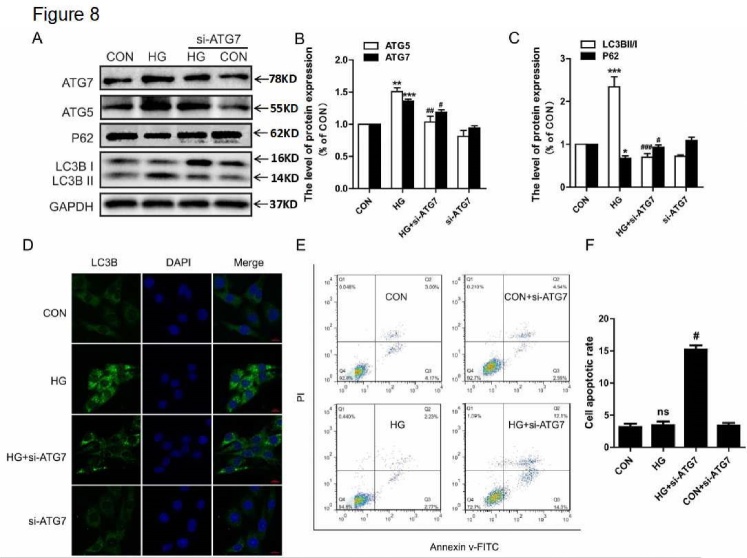
ATG7 siRNA treatment abolished the neuroprotective role of autophagy under HG conditions. (A) Western blot analysis of ATG7, ATG5, P62, and LC3B expression. (B) Quantitative analysis of ATG5 and ATG7. (C) Quantitative analysis of LC3B and P62. (D) Immunofluorescence staining of LC3B in PC12 cells. (E) The results of flow cytometry under different conditions. (F) The level of apoptosis in PC12 cells. CON: control, HG: high glucose. Scale bar = 50 μm. *P < 0.05 *vs.* CON, **P < 0.01 *vs.* CON, ***P < 0.001 *vs.* CON, #P < 0.05 *vs.* HG group, ##P < 0.01 *vs.* HG group, ###P < 0.001*vs.* HG group, and N = 3.

To further confirm the neuroprotective role of autophagy under HG conditions *in vitro*, the PC12 cells were treated with ATG7 siRNA. We found that si-ATG7 inhibited the expression of ATG7, ATG5, and LC3B-II, and it decreased the degradation of P62 under HG conditions (Fig. 8A-C). Immunofluorescence results also showed that si-ATG7 treatment attenuated the HG-induced increase in LC3B puncta (Fig. 8D). Then, we examined the effect of si-ATG7 on cell apoptosis by flow cytometry. Under normal glucose conditions, si-ATG7 had no significant effect on cell apoptosis when compared to the CON group (3.40 ± 0.72% versus 3.15 ± 0.92%, Fig. 8E and F). However, ATG7 siRNA treatment significantly increased cell apoptosis under HG conditions when compared to the CON group (15.18 ± 1.16% versus 3.46 ± 0.97%, Fig. 8E and F).

## DISCUSSION

Diabetes is a chronic metabolic disorder that is characterized by hyperglycemia and insulin resistance. It is involved in series of complications, such as hypertension, renal disease, and nervous system diseases. Increasing evidence suggests that diabetes is associated with cognitive decline, and the level of hyperglycemia and duration of diabetes have been shown to be linked with cognitive decline[29-31]. The hippocampus is an important functional area of the brain that is involved in short-term memory, learning, executive ability, and attention. It is divided into three regions: CA1, CA3, and dentate gyrus (DG). The CA1 and CA3 region of the hippocampus are most closely related to learning and memory, especially CA1. CA1 is a vulnerable area of the hippocampus under conditions of ischemia, hypoxia, and other damaging stimuli [32]. It has previously been reported that diabetes influences the structure and function of neurons, axons, and synapses in CA1 region. This then changes synaptic plasticity, which subsequently leads to the decline of LTP and affects the learning and memory processes [33]. Thus, we focused on the effect of diabetes on CA1 and CA3. Consistent with previous studies, the present study confirmed that diabetes induces the accumulation of Aβ_1-42_ in hippocampal CA1 and CA3 and reduces the expression of BDNF and MAP2. Our results have confirmed that diabetes impaired cognitive function with inferior learning and memory function.

Autophagy is an essential process for the maintenance of cellular homeostasis in the central nervous system under physiological and pathological conditions [34,35]. A growing body of evidence suggests that autophagy contributes to the obliteration of accumulated cytotoxic protein in the brain. This would have a neuroprotective role on several neurodegenerative diseases, including AD and PD [36-38]. In the current study, we found that diabetes significantly increased autophagic activation, as evidenced by the upregulation in the expression of LC3B-II, ATG5, and ATG7 and the downregulation of P62 in the hippocampus. *In vitro* treatment with an autophagy inhibitor, 3-MA, or si-ATG7 significantly induced the level of apoptosis under HG conditions. These findings suggest that autophagy may play a protective role in DACD.

It has been shown that diabetes significantly impairs the cognitive function of mice with inferior learning and memory. This implies that the neuroprotective role of autophagy in DACD is limited. We speculate that the increased activation of autophagy during DACD is an acute response of damaged neurons to toxic substances, such as the accumulation of abnormally folded proteins, impaired mitochondria, and reactive oxygen species (ROS). Previous studies have reported that mitochondria-mediated microtubule conformational changes caused axonal transport barriers; thus, autophagy vesicles are not able to fuse with the lysosome and vesicle in the axon[39]. It is well known that LC3B-II, ATG5, ATG7 and P62 are involved in the initiation and extension of autophagosome formation. Our *in vitro* results revealed that autophagic activity was gradually induced after 12 hr or 24 hr of treatment with HG, but it was decreased at 48 hr. Diabetes induces cell apoptosis, which is a causal event among many diabetes-associated complications [40,41]. Accompanying changes in autophagy, the level of apoptosis was not significantly affected at 12 hr and 24 hr, but it was upregulated at 48 hr. These results indicate that autophagy may play a neuroprotective role at the early stage of DACD. However, the cross-talk between autophagy and apoptosis during DACD needs to be further studied.

The accumulation of misfolded proteins is a characteristic of age-related neurodegenerative diseases. The endoplasmic reticulum (ER) plays a vital role in protein synthesis, folding, calcium, and homeostasis. Physiological or pathological conditions that perturb these processes result in elevated ER stress. Diabetes significantly triggers ER stress that leads to excessive apoptosis. This is implicated in diabetes-related complications, such as retinopathy and nephropathy [42,43]. Whether there is cross-talk between ER stress and autophagy during DACD is still unclear. A recent study demonstrated that inhibition of ER stress reversed AGEs-induced autophagy in mesangial cells, but autophagy inhibition did not influence AGEs-induced ER stress[44]. Additionally, diabetes influenced the expression of ER stress-related genes in the hippocampus, and hippocampal cells adapted to prolonged ER stress induced by T2D with partial suppression of Xbp1[45]. Thus, we speculate that inhibition of ER stress is involved in the neuroprotective role of autophagy during DACD, but this needs to be further elucidated in future studies.

Generally, astrocytes are considered to support and nourish neurons in the central nervous system. Recently, increasing evidences have demonstrated that astrocytes are also involved in neurotransmitter release in the presynaptic neuron, which receives and integrates the neural signals from multiple adjacent neuron synapses [46,47]. It has also been reported that astrogliosis contributes to diabetes-associated memory impairment [48]. Using the metabonomics analytical method, it was also revealed that diabetes induces astrocyte proliferation and neuronal apoptosis in the hippocampus, and it disturbs energy metabolism and the glutamate-glutamine cycle between astrocytes and neurons[49]. The current study indicates that autophagy plays a neuroprotective role during DACD. It is well known that autophagy could be induced by a limited availability of ATP or a lack of essential nutrients, such as glucose and amino acids, and the accumulation of specific metabolites or metabolic byproducts [50]. Thus, it is speculated that autophagy may be involved in the regulation of metabolism alteration between astrocytes and neuron in the hippocampus during DACD.

In conclusion, our study has confirmed that diabetes significantly increased cognitive decline with impeded learning and memory function. We also demonstrated that autophagy activation has a neuroprotective role in DACD, suggesting that targeting autophagy may be a potential therapeutic strategy for the treatment of DACD. However, the molecular mechanism underlying the neuroprotective role of autophagy in DACD is still unclear, which needs further study.
